# Mechanisms of Epigenetic Inheritance in Post-Traumatic Stress Disorder

**DOI:** 10.3390/life14010098

**Published:** 2024-01-08

**Authors:** Pei-Chen Chou, Yu-Chi Huang, Sebastian Yu

**Affiliations:** 1 School of Medicine, College of Medicine, Kaohsiung Medical University, Kaohsiung 80708, Taiwan; u110001005@gap.kmu.edu.tw; 2 Department of Psychiatry, Kaohsiung Chang Gung Memorial Hospital and Chang Gung University College of Medicine, Kaohsiung 83301, Taiwan; ychuang01@gmail.com; 3 Department of Dermatology, College of Medicine, Kaohsiung Medical University, Kaohsiung 80708, Taiwan; 4 Department of Dermatology, Kaohsiung Medical University Hospital, Kaohsiung 80756, Taiwan; 5 Master of Public Health Degree Program, National Taiwan University, Taipei 10617, Taiwan; 6 Neuroscience Research Center, Kaohsiung Medical University, Kaohsiung 80708, Taiwan

**Keywords:** DNA methylation, histone modification, intergenerational epigenetic, maternal inheritance, non-coding RNA, paternal inheritance, transgenerational epigenetic

## Abstract

Post-traumatic stress disorder (PTSD) is a psychiatric disorder that causes debilitating functional impairment in patients. Observations from survivors of traumatic historical events solidify that this disease is not only associated with personal experiences but can also be inherited from familial traumas. Over the past decades, researchers have focused on epigenetic inheritance to understand how responses to adverse experiences can be passed down to future generations. This review aims to present recent findings on epigenetic markers related to PTSD and research in the intergenerational inheritance of trauma. By understanding the information, we hope that epigenetic markers can act as biochemical measurements for future clinical practice.

## 1. Introduction

Environmental factors have been attributed to the individual’s susceptibility to psychiatric disorders [1], and the underlying mechanisms have been under extensive research. One of the psychiatric diseases that is greatly subjected to environmental stimuli is post-traumatic stress disorder (PTSD). PTSD is a trauma- and stressor-related disorder with the required criteria of exposure to traumatic events before the development of related symptoms [2]. The trauma events, including not only a repercussion of catastrophic life experiences such as a past or ongoing war, physical or sexual trauma, but also schoolyard and workplace bullying [3] as well as adverse childhood experience [4], could also serve as disease triggers [5]. To understand how the effect of environmental triggers persist throughout one’s lifetime, over the past decades, scientists have turned to epigenetics in an attempt to look for interactions between the environment and the individuals in which it immerses.

Epigenetics can be simply described as transcriptional regulation which subsequently alters the resulting phenotype of the organism [6]. In other words, epigenetic processes do not interfere with the DNA code itself but make changes in the chromosome and the expression level of mRNA transcripts [6]. This allows for an organism to make immediate responses to the environment, without permanently changing its genetic making [6]. However, this does not mean that epigenetic changes can only be temporary; when an individual is exposed to a certain environmental stressor for the appropriate amount of time, epigenetic changes persist for the body to accommodate the new norm [6].

The inheritance of both physiological and psychological stress through epigenetic mechanisms has been extensively studied in the past few decades. From the keen observations on offspring of Dutch famine survivors, periconception exposure to physiological stress (starvation) has been first found to contribute to long-lasting hypomethylation in the IGF2 DMR gene for human development [7]. In recent decades, increasing literature on the epigenetics of psychological stress emerged. Researchers have also observed epigenomic differences in offspring of devastating historical events, including but not limited to the Holocaust [8], maltreatment toward First Nations in Canada [9], Quebec ice storm [10], and the 9/11 World Trade Center terrorist attack [11]. Therefore, we will elaborate on the epigenetic impact of traumatic experiences on future generations, as well as current skepticism on epigenetic inheritance.

This review article has four aims: starting with providing background information on epigenetic mechanisms, we will present updates on recently discovered epigenetic markers of psychological stress; furthermore, we collate human studies on the inheritance of trauma utilizing epigenetics; finally, we end with suggestions on directions for future studies in the hope of providing the readers a comprehensive understanding on this growing field of biological psychiatry.

## 2. Epigenetic Mechanisms

To promptly react to environmental changes and increase fitness, the expression of genes is under epigenetic control. Epigenetic mechanisms modify the level of transcripts that could subsequently proceed to translation. These changes are flexible yet durable, as they could be changed within a cell cycle and maintained throughout a lifetime [6]. Some of the epigenetic mechanisms commonly used in the human body are DNA methylation, histone modifications, and non-coding RNAs.

### 2.1. DNA Methylation

DNA methylation is the most studied epigenetic mechanism of all. It was originally discovered in 1944 by Hotchkiss. In the 1980s, it was confirmed that methylated cytosine is involved in gene expression [12]. DNA methylation typically functions as a repressor of transcription of the modified gene, especially when it occurs in the promoter region [13]. It plays a critical role in processes such as silencing retroviral elements, regulating tissue-specific gene expression, imprinting, and X chromosome inactivation [12,14]. The entire process, from placing and removing markers to the recognition of DNA silencing, requires a writer, an eraser, and a reader. The methylation of DNA is regulated and maintained by the DNMT (DNA methyltransferases) family (DNMT, DNMT3a, DNMT3b, and DNMTl) typically on the C5 (carbon number 5) position of cytosines that are followed by a guanine nucleotide; such sites are termed CpG sites [12]. Although the majority of methylations occur on CpG sites, methylations have also been found on cortices non-CpG (CpC, CpA, CpT) sites [12]. The existence of a single demethylation enzyme remains debatable; however, a consensus has been reached on the involvement of Tet enzymes and base excision repair. The ten–eleven translocation (Tet) enzymes (Tet1, Tet2, and Tet3) catalyze the formation of the 5hmC (5-Methylcytosine) intermediate, which could then be converted via further hydroxylation by Tet and deamination by AID/APOBEC to molecules (Thy, 5hmU, 5fC, and 5caC) that will be excised by BER and replaced by a cytosine. Finally, the methylated cytosines are read by the MBD (methyl-CpG binding) proteins, the UHRF (ubiquitin-like, containing PHD and RING finger domains) proteins, and the zinc-finger proteins to silence the expression or reinforce the methylation during replication. As one can infer from its role, the CpG site undergoes extensive differential methylation during gametogenesis and embryonic development. DNA methylation dysregulation is associated with numerous diseases, including Rett syndrome, hereditary sensory and autonomic neuropathy type 1 (HSAN1), Prader–Willi Syndrome, and Angelman Syndrome.

### 2.2. Histone Modification

Histones are protein molecules that provide the scaffold for DNA to be organized into nucleosomes and later into chromatins, and the modifications on histones will therefore affect chromatin structure as well as the recruitment of nucleosome-remodeling enzymes. Histone modification primarily includes histone (de)acetylation, histone methylation, and histone phosphorylation. Histone acetylation on lysine residues is catalyzed by histone acetyltransferase (HAT). Lysines on both the histone core and tail could be acetylated. Acetylated lysine has a weaker affinity to the DNA molecule, allowing for it to be easily accessible by transcriptosomes for transcription. Deacetylation of lysine by histone deacetylases (HDAC) creates effects opposite to those of histone acetylation, as it condenses the chromatin, repressing gene expression. Lastly, serine, threonine, and tyrosine residues, predominantly those on the histone tail, can undergo histone phosphorylation. A few kinases responsible for histone phosphorylation have been identified, as well as their antagonizing phosphatases [15].

### 2.3. Non-Coding RNAs (ncRNA)

The final epigenetic processes we will be discussing are non-coding RNAs (ncRNA). Only a fraction of the human transcriptome encodes protein [16]. Each class of RNA has unique cellular functions, some of which are types of machinery modulating gene expression levels by chromatin remodeling at the transcriptional or post-transcriptional level [16]. RNAs with epigenetic functions include micro RNA (miRNA), small interfering RNA (siRNA), promoter-associated RNA (PAR), enhancer RNA, and long non-coding RNA [17]. The most abundant ncRNA is the lncRNA. MiRNA and siRNA regulate the expression of around 50% of human genes by binding to and cleaving complementary mRNA transcripts [17]. PAR has been found to have repressive or activation effects in different studies [17]. When associated with the polycomb protein group, PARs exhibit a repressive function. As their name suggests, the enhancer RNAs activate the transcription of genes. LncRNA are the most abundant of all ncRNA, and they recruit chromatin-remodeling proteins [17]. However, since ncRNA asymmetrically segregates into the daughter cells, its role as an epigenetic marker has been under debate [18].

## 3. Identifying Epigenetic Markers of PTSD

The epigenome-wide association study (EWAS) is the most recent approach to identifying epigenetic markers. Analogous to the genome-wide association study (GWAS) method, which assesses the entire genome for disease association, EWAS aims to assess whole-genome epimutations about diseases, without a hypothesis. Despite many epigenetic mechanisms, due to technological restrictions, most EWAS to date have been focusing on DNA methylations [19]. Compared to GWAS, an ideal EWAS design requires a more deliberate selection of experimental and control samples as well as statistical corrections to account for the dynamic epigenome [20] that varies between populations. The whole-genome data of methylation sites are then obtained from the tissue of interest through various typing and profiling technologies [21], and differences between groups can be compared in a site-specific, CpG-cluster, or regional fashion after the aforementioned statistical corrections [22].

Much like genome-wide association studies, the EWAS method [23] commonly acquires methylome data from peripheral blood samples instead of the tissue of interest (i.e., brain tissue for PTSD), considering the accessibility and simplicity of study design. However, controversy surrounds such a choice of sample tissue, due to the tissue-specific nature of epigenetic markers [20]. All cells in the human body share the same genome, and thus obtaining genomic data from blood samples for GWAS is deemed to be appropriate [20].

Noticeably, the methylome and epigenome do not exhibit uniformity across different tissues [20]. Cells in different tissues perform their distinct tasks and maintain expression patterns through cycles of cell division using epigenetics [20]. Therefore, the resulting data should be interpreted carefully, keeping in mind the epigenetic differences between blood cells and the experiment’s cell of interest [20]. Similarly, the tissue-specific nature of epigenetic markers also requires careful comparison, commonly with statistical analysis, between the acquired data and original epigenetic markings of the discussed tissue to ensure the identified deviation as epimutation [20].

Despite the hardship of conducting epigenetic studies, many advancements have been made in identifying PTSD-related DNA methylation sites. Epigenetic changes in genes participating in physiological processes such as the HPA axis, immune function, neuroplasticity, circadian rhythms, and cell adhesion are of particular interest.

Specifically, PTSD has been linked to dysregulation of the hypothalamus–pituitary–adrenal axis (HPA axis). This stress response pathway develops in early fetal stages [9] and is easily affected by the environment. When the HPA axis is incapable of returning to a normal physiological state, a prolonged state of stress occurs. Among the many genes involved in the HPA axis, two of them are most extensively studied: NCR3C1, the glucocorticoid receptor gene, and FKBP5, the co-chaperone that inhibits glucocorticoid receptor (NR3C1 product) function [24,25,26]. Multiple CpG sites on variants of these genes have been found to contribute to PTSD symptoms and resilience to trauma [26].

Interestingly, methylation on the cg19645279 site on the NR3C1 gene and the cg07485685 site on the FKBP5 is found to be associated with both symptom severity and resilience.

Other methylation differences related to PTSD and trauma include the BRSK1 (tumor suppressor), LCN8, NFG, DOCK2 (immune cells chemotaxis), ZFP57(transcriptional repressor), RNF39 (synaptic plasticity), NRG1(cell–cell signaling), HGS (lysosome-dependent degradation) genes, MAN2C1 (apoptosis regulation), TPR (cellular trafficking), ANXA2 (signal transduction and cellular growth), CLEC9A (myeloid cells activation), ACP5 (glycoprotein), TLR8 (pathogen recognition), CXCL1 (chemoattractant of immune cells) and BDNF(neuroplasticity) [25,27,28]. Differential expressions of these genes are not only associated with PTSD but also cause comorbidity of other psychiatric diseases.

In a large-scale EWAS in 2020, Logue et al. [21] identified ten new methylations that are strongly associated with PTSD and genes that confirmed previous findings. Some of these genes and their functions are noteworthy for future PTSD studies, including G0S2 (lipid metabolism, downregulated in response to stress), APBA1/2 (protein transportation and synaptic function in brain tissue), CHST11(extracellular signaling and neuronal plasticity in brain tissue), and AHRR (xenobiotic metabolism) [29]. A recent study investigating 10 cohorts discovered hypermethylation in the AHRR gene, these methylations are independent of cigarette-smoking status, and the researchers elaborated that such changes could explain the association between PTSD and immune dysregulation [30].

Another study performed on American military cohorts suggests differential SPRY4 methylation, long non-coding RNA SPRY4 expression (associated with suicidal behavior), MAD1L1 (mitotic spindle-assembly checkpoint), and HEXDC gene expression [25,31].

Two studies in 2021 narrowed their subject of interest to female sexual violence survivors. An analysis focusing on female rape survivors identified epigenetic markers specifically associated with PTSD triggered by sexual trauma [32,33]. On top of NR3C1 hypermethylation [34,35], statistically significant results have been found in the CpG site cg01700569 close to the SLC16A9 gene, although the protein is less known to be associated with mental disorders. Aligning with previous studies, participants with PTSD are found to have decreased BRSK2 methylation at 3 months post-rape, and similar results have been found in BRSK1 methylation in other PTSD cohorts. Both the BRSK2 and BRSK1 are expressed in the hippocampus, a brain region associated with trauma, memory, and the fight-and-flight response. Also consistent with the other PTSD research, the decrease in AHRR methylation (at cg05575921 and cg26703534) remained significant in this cohort. Another similar study on war-time sexual violence survivors discovered differential methylation patterns in SLC6A, OXTR promoter, and NINJ2 [33]. Epigenetic modifications relating to PTSD are summarized in Table 1.

## 4. Intergenerational and Transgenerational Inheritance of Epigenetic Markers

The identification of epigenetic markers is a big step towards understanding the non-genetic cause of PTSD. Furthermore, genetic or epigenetic changes made in the germline indirectly affect the epigenetics of future generations. Nonetheless, when the epigenetic effect is observed in later generations, it could be passed down via multiple routes which are classified into two categories: intergenerational inheritance and transgenerational inheritance [37]. Even though some researchers consider these two terms to be interchangeable, we feel that it is beneficial to differentiate these inheritance processes according to Skinner’s definition. Please note that in the following sections, when referring to transgenerational/intergenerational inheritance studies, corrections will be made according to Skinner’s definition [37].

**Figure 1:**
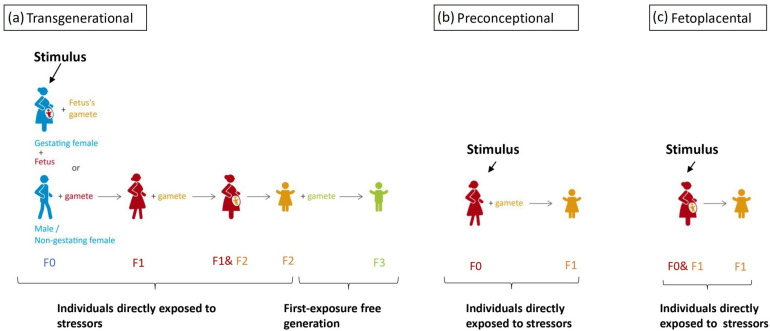
A color-coded schematic drawing of stimulus application timing of each route intergenerational inheritance, with the same color indicating the same individual. (**a**) For true transgenerational inheritance, stress response should be recorded in the first generation that is not directly affected by the stressor. If a fetus is exposed to stressors during pregnancy, we cannot exclude the epigenetic changes in F1 are independent of changes in F0. Therefore, for male/non-gestating female F0, a response should be recorded in the F2 generation. However, for gestation female F0, stressors directly affected the F0, F1, and F2 generation; therefore, a response would have to be recorded in the F3 generation. (**b**,**c**) Preconceptional and fetoplacental inheritance both concerns 2 generations to which the is stressor directly applied, the F0 and the F1 (the gamete or the fetus of F0).

Intergenerational inheritance implies a transmission process between two generations [38]. As the gamete of the trauma-exposed F0 generation is already present during the exposure, the definition of intergenerational inheritance does not rule out the possibility of direct traumatic exposure of F1 genetic material within the gamete, embryo, and fetus. This means of inheritance is widely studied due to the high malleability of the epigenome during embryogenesis. Transgenerational epigenetic inheritance has a more rigorous criterion based on biological sex and pregnancy status. The affected individual: when the affected individual is male or a non-gestating female, the environmental stressor affects both the individual and their germline cells and, in such cases, the epigenetic change must persist to the third generation to be considered transgenerational inheritance [39]. In gestating females, however, germline cells developing withing within the fetus could also be directly affected by the stimuli [37]. Therefore, any stressor applied is considered a direct stressor to all three generations [40], which renders the next generation to be the first without direct traumatic experience. In short, transgenerational inheritance is only said to occur when the epigenetic marker is present in the first exposure-free generation to the original stressor; this criterion ensures that epigenetic markers are transmitted. For the above reasons, preconception trauma in women, fetoplacental interactions, and improper parental care are considered to be intergenerational inheritance [41] and shall not be confused with the transgenerational inheritance of trauma, as in these scenarios the subject of interest receives direct traumatic experience. The concept of transgenerational, preconceptional, and fetoplacental inheritance is illustrated in Figure 1.

## 5. Different Timings of Trauma Exposure and Intergenerational Inheritance Studies

There has been compelling evidence suggesting physiological changes in the offspring of parents who experienced traumatic events. In 2018, Yehuda et al. reviewed human studies of the epigenetic intergenerational inheritance of stress [9]. The routes of intergenerational inheritances were categorized by the time of trauma exposure: maternal care (post-natal exposure), fetoplacental interaction (in utero exposure), preconception trauma (gamete exposure), and transgenerational inheritance. Differential findings might suggest that inheritance routes play a significant role in stress inheritance. For the above reason, we will report the latest findings of human epigenetic inheritance classified based on the route of transmission.

### 5.1. Post-Natal Exposure

Although perturbation caused by insufficient parental care should not be deemed as a means of inheritance, mood disorders can impair the interaction between the patient and their offspring, acting as a form of childhood adversity [42]. Adverse childhood experiences (ACE) have a large impact on the neural development of an individual. ACEs have been associated with psychiatric diseases such as PTSD, anxiety, depression, and bipolar disorder, as well as physical illnesses such as diabetes and cardiovascular diseases. Diseases are caused by differential methylation patterns on genes relating to neurotransmission, specifically the decrease in methylation on FKBP5 (heat shock protein for GR) and MAOA (degradation of monoamine neurotransmitter; also, the increase in methylation on NR3C1 (GR gene), HTR (serotonin receptor), SLC6A4 (serotonin reuptake transporter), and BDNF (promote neuroplasticity)) [43]. On top of these findings, it has been found that maternal overprotection can also cause alterations in hormone expression in holocaust survivors [44]. The complexity of human parenting style has also added to the complication of mood regulation studies.

### 5.2. In Utero Exposure

Experiences as early as in utero exposure have been found to contribute to PTSD vulnerability through DNA methylation [45]. Increased methylation has been found in the promoter region of the NR3C1/2 gene in both mothers (F0) who were pregnant during the Tutsi genocide and their children (F1) [9,46]. Similar findings have also been found in women pregnant while experiencing domestic violence, in the war zones of the democratic republic of Congo [34], during the Rwanda genocide [46], and the Quebec ice storm. A study on women who experienced sexual violence showed expression in F1 through fetal placental differential methylation patterns on the SLC6A4INJ2, OXTR promoter, and NINJ2 genes and related regions [33].

### 5.3. Preconceptional Inheritance and Transgenerational Inheritance

Among all routes of epigenetic inheritance of stress, preconceptional trauma inheritance and transgenerational inheritance are particularly under debate [47]. This is based on the understanding that global reprogramming of the epigenome occurs in the mammalian germline immediately after fertilization to erase acquired epigenetic marks or epigenotype [14,48]. Although retention has been found in methylation in some regions of the genome, the sperm histone PTM, and sperm small ncRNAs, more studies are required to understand the mechanism behind the preservation of epigenetic markers [39]. Still, observations of epigenetic changes in later generations provide evidence that some methylations exhibit meiotic stability [49].

#### 5.3.1. Preconceptional Inheritance

Maternal preconception stress can exert an effect on the oocytes as well as the uterine environment, and one should keep in mind that it is difficult to discern the route of transmission even when epimutations are present in both F0 and F1. However, there have been some studies which focus on the relationship between maternal stress and offspring stress regulation. In holocaust survivors, associations have been found between F0 stress and lower FKBP5 methylation in offspring.

Recently, several studies have suggested paternal epigenetic inheritance during spermatogenesis [50]. This style of transmission has not received great attention in the early stages of epigenetic research [51]. Paternal transmission guarantees the absence of fetoplacental interactions and maternal care factors [9]. In 2011, it was determined that miRNA, piRNA, and sncRNA within mature human sperm can act as vertical information carriers and key regulators of transgenerational inheritance of gene expression [52,53,54]. Histone modifications that are retained through spermatogenesis have also been found to be crucial in embryo development [48]. An EWAS performed by Mehta et al. in 2019, and it identified DNA methylation marks of veterans’ peripheral blood and spermatozoa, attempting to find overlapping patterns relating to PTSD development and heritability [25]. They have discovered three CpG sites on the CCDC88C associated with PTSD severity in the genome of sperm. They have also identified an association between 10 CpG sites and PTSD diagnosis within the offspring of the veterans [25]. However, without data on the offspring’s epigenome, it is uncertain if the offspring’s PTSD was transmitted through the germline. The evidence is also insufficient to conclude that methylation could persist through fertilization [25].

#### 5.3.2. Transgenerational Inheritance

Unfortunately, to our knowledge, there are few studies on human transgenerational epigenetic inheritance of PTSD. Human studies on this topic suffer from limitations such as the generation required for effects to be observed, confounding factors, and ethical issues [55]. Without transgenerational inheritance evidence, it is uncertain if these biological memories can be passed down through the germline. Nevertheless, it is observed that the F2 offspring of holocaust survivors (F0) suffer from psychological impairment. These symptoms could be due to the increase in FKBP5 expression and decrease in NR3C1 methylation, providing insights into epigenetic transmission across three generations [1].

## 6. Cultural Level PTSD

Despite uncertainties revolving around the level of demethylation and re-methylation during fertilization, it is still exciting to discover epigenetic changes in succeeding generations. As compelling evidence of transgenerational epigenetic inheritance of PTSD is brought to light, the alarming subject of epigenetic changes in an entire generation due to cultural trauma has been broached. In 2018, Ching et al. reviewed studies of PTSD in people of color and theorized that racial trauma across several generations can have cumulative epigenetic effects [56]. Disturbingly, racial traumas that are associated with PTSD are not uncommon in day-to-day life, including workplace discrimination, the use of racial slurs, and immigration difficulties.

## 7. Future Directions and Use

The current psychiatric diagnosis described in the DSM-5 or ICD-11 relies on the psychiatrist’s diagnostic interview for assessing if the individual’s mental status meets the corresponding listed diagnostic criteria at the time of the medical visit. However, the categorical system of diagnosis criteria and the patient’s memory recall bias contribute to diagnosis heterogeneity [57,58]. The easily accessible biological markers help to optimize precision in the clinical diagnosis of psychiatric diseases [59]. The epigenetic markers of PTSD on somatic cells may allow for scientists to access a patient’s experience and family history. Epigenetic data can be obtained from human and cadaver cells, suggesting the application of this evidence in the legal system [60]. Furthermore, although most studies have focused on the detrimental effect of trauma inheritance, not all epigenetic markers cause pathology, and some are associated with resilience. In a small pilot study, Miller et al. discovered that methylations in different sites of the FKBP5 and NR3C1 could have positive contributions toward mental health, such as increasing the subject’s resilience [24]. Recently, studies have shown that high-quality parental care results in increased histone acetylation in hippocampal tissue, decreased methylation of N3CR1 and BDNF, and increased methylation of TNF. These epigenetic changes are associated with reduced psychological pathology [55]. We suggest studies with larger sample sizes be conducted on epigenetic changes that could improve resilience. This knowledge could be applied to improve the psychological health of workers in particularly stressful workplaces, such as military personnel and nurses [3].

The revelation of epigenetic markers and their effects sheds light on the prospect of epigenetic inheritance of resilience, sparking subsequent studies to have led to later studies on their suitability as diagnostic biomarkers [55]. NR3C1 overexpression and HPA- axis-related methylation changes in PTSD have been proposed to be used as a diagnostic marker to assess PTSD treatment efficacy [61]. Most recently, in 2023, Wilker et al. found that methylation on the cg25535999 CpG site of the NR3C1 intron is negatively associated with PTSD symptoms (however, this study lacks data on GR expression level) [62]. Other epigenetic markers have been proposed to be associated with recovery or the prediction of treatment response [36,63,64,65,66,67,68,69]. Lastly, in 2021 Vinkers et al. revealed the possibility of reversing DNA methylation at the ZFP57 gene as a treatment for PTSD, opening the window to a new method of PTSD and psychiatric disorder treatment via epigenetics [36].

## 8. Closing Statements

For a long time, genetic research has been the focus of scientific research and the main understanding by laypersons of many psychiatric phenomena, such as criminal behavior, political participation, or sexual orientation [70]. Now, nature and nurture are no longer at two ends of the tug-of-war, competing for the cause of psychiatric diseases, but two interwoven ideas. Epigenetics inheritance demonstrates that the effects of environmental stimuli can be written into heritable material and could even persist to the next generation. These inherited traits prime the future generation for their response to more environmental stimuli. Furthermore, discoveries in the epigenetic field have further opened our eyes to new ways of diagnosing, understanding, and treating psychiatric diseases.

In this review, we have presented basic knowledge and recent research regarding epigenetics’ contribution to PTSD. The advancements in this field provide a strong background for discovering more epigenetic markers and environmental stimuli relevant to PTSD; they have also shined light on the epigenetic research of other psychiatric phenomena.

## Figures and Tables

**Table 1:** Table summary of mentioned epigenetic modifications relating to PTSD.

Gene	Gene Function	Modification	Modification Site	Reference
ACP5	glycoprotein	Methylation	-	[27]
AHRR	xenobiotic metabolism	Methylation	-	[30]
AHRR	xenobiotic metabolism	Demethylation	cg05575921 and cg26703534	[33]
ANXA2	signal transduction and cellular growth	Methylation	-	[27]
APBA1/2	protein transportation and synaptic function in brain tissue	Methylation	-	[29]
BDNF	neuroplasticity	Methylation	-	[27]
BRSK1	tumor suppressor	Methylation	-	[27]
CCDC88C	coiled-coil domain containing 88C	Methylation		[25]
CHST11	extracellular signaling and neuronal plasticity in brain tissue	Methylation	-	[29]
CLEC9A	myeloid cells activation	Methylation	-	[27]
CXCL1	chemoattractant of immune cells interacting with BDNF to regulate neuroplasticity	Methylation	-	[27]
DOCK2	immune cells chemotaxis	Methylation	-	[27]
FKBP5	co-chaperone inhibiting glucocorticoid receptor	Methylation	cg07485685	[25,27,28]
G0S2	lipid metabolism, downregulated in response to stress	Methylation	-	[29]
HGS	lysosome-dependent degradation	Methylation	-	[28]
HEXDC	enables beta-N-acetylhexosaminidase activity	Methylation		[31]
LCN8	ligand transportation	Methylation	-	[27]
MAD1L1	component of the mitotic spindle-assembly checkpoint	Methylation		[31]
MAN2C1	apoptosis regulation	Methylation	-	[27]
NCR3C1	glucocorticoid receptor	Methylation	cg19645279	[25,34,35]
NRG1	cell–cell signaling	Methylation	-	[28]
RNF39	synaptic plasticity	Methylation	-	[28]
SPRY4	associated with suicidal behavior	Long non-coding RNA	-	[29]
TLR8	pathogen recognition	Methylation	-	[27]
TPR	cellular trafficking	Methylation	-	[27]
ZFP57	transcriptional repressor	Methylation	-	[28,36]

## Data Availability

Not applicable.
